# Serial dependence in face-gender classification revealed in low-beta frequency EEG

**DOI:** 10.1186/s12915-025-02289-6

**Published:** 2025-07-08

**Authors:** Giacomo Ranieri, David C. Burr, Jason Bell, Maria Concetta Morrone

**Affiliations:** 1 Department of Neuroscience, Psychology, Pharmacology, and Child Health, University of Florence, Florence, 50135 Italy; 2 School of Psychology, University of Sydney, Camperdown, 2050 Australia; 3 School of Psychology, University of Western Australia, Crawley, 6009 Australia; 4 Department of Translational Research On New Technologies in Medicine and Surgery, University of Pisa, Pisa, 56123 Italy

**Keywords:** Face-gender discrimination, Serial dependence, EEG decoding, Vision, Beta oscillations

## Abstract

**Background:**

Perception depends not only on current sensory input but is also heavily influenced by the immediate past perceptual experience, a phenomenon known as “serial dependence,” particularly robust in face perception.

**Results:**

We measured discrimination of face-gender in participants to a sequence of intermingled male, female, and androgynous images, while recording EEG responses. The discriminations showed strong serial dependence (androgynous images biased towards male when preceded by male and female when preceded by female). The strength of the bias oscillated over time in the beta range, at 14 Hz for female prior stimuli, 18 Hz for male. Using classification techniques, we were able to successfully classify the previous stimulus from current EEG activity. Classification accuracy correlated well with the strength of serial dependence across individual participants, confirming that the neural signal from the past trial biased face perception. Bandpass filtering of the signal within the beta range showed that the most useful information to classify gender was around 14 Hz when the previous response was “female,” and around 18 Hz when it was “male,” reinforcing the psychophysical results showing serial dependence to be carried at those frequencies.

**Conclusions:**

Overall, the results suggest that recent experience of face-gender is selectively represented in beta-frequency (14–20 Hz) spectral components of intrinsic neural oscillations.

**Significance statement:**

The neurophysiological mechanisms of how past perceptual experience affects current perception are poorly understood. Using classification techniques, we demonstrate that the response to gender of the previous face image of a sequence can be decoded from the neural activity of the current EEG response, showing that relevant neural signals are maintained over trials. Classification accuracy was higher for participants with strong serial dependence, strongly implicating these signals as the neural substrate for serial dependence. The best information to classify gender was around 14 Hz for “female” faces, and around 18 Hz for “male,”, reinforcing the psychophysical results showing serial dependence to be carried at those beta -frequencies.

## Background

Much evidence has accumulated to suggest that the brain is a prediction machine, which generates perceptual experience from internal models based on previous perceptual experience [1–5]. On this view, the brain must update predictions to minimize the discrepancy between internal models and external stimuli, in a constantly changing environment. Recent research suggests that low-frequency neural oscillations are a candidate for the role of messengers of top-down predictions [6–10]. Alpha and low-beta (up to ~ 18 Hz) oscillations seem to be firmly implicated in transmitting recent history, both for maintenance of working memory [11–14] and in transmitting perceptual *priors* [15, 16].

Perception of faces—including identity [17], gender [18], and attractiveness [19]—are all strongly susceptible to *serial dependence*, a phenomenon in which the current percept is systematically biased towards that of recently seen stimuli [5, 20]. Bell et al. [21] further showed that beta oscillations are strongly implicated in the serial-dependence induced bias of gender, and that the oscillation frequency differs between male and female images: faster for male biases (17 Hz) and slower for female biases (~ 13 Hz).

Motivated by these psychophysical findings, we investigated the neurophysiological characteristics of perceptual history information embedded within low-frequency neural oscillations, using classification techniques of electroencephalogram (EEG) recorded from participants during a face-gender discrimination task.

## Results

### Behavioral results (serial dependence and oscillations in bias)

**Fig. 1:**
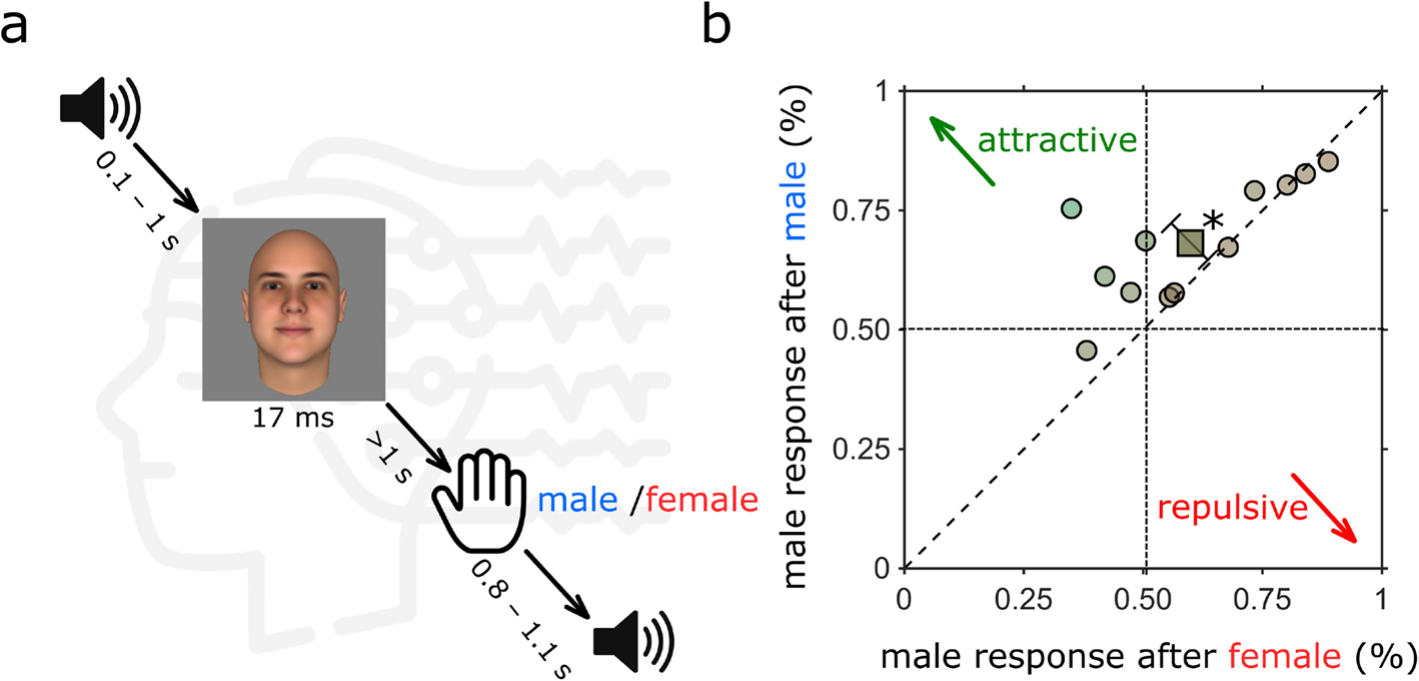
Experimental paradigm and behavioral results. **a** Schematics of the experimental paradigm. Each trial started with an audio cue followed, after a random interval (between 100 and 1000 ms), by a brief (16.6 ms) face presentation. There were 15 different face identities, each morphed into 3 genders (androgynous, female, and male), making 45 faces in total. Participants waited at least 1 s before responding by keypress, indicating whether the face appeared more female or male. A new trial started 800 to 1100 ms after the response. **b** Proportion male responses to androgynous stimuli, depending on the response to the previous stimulus (response “female” plotted on abscissa, “male” on ordinate). Gray circles represent individual participants. The average bias towards *male* is given by the distance of the datapoint projection along the positive diagonal, serial dependence (towards or away from the previous stimulus) by the distance of the point to that diagonal. Data for most participants fall above the equality line (tending to respond more male if the previous was male, and vice versa), evidence for attractive serial dependence. The average serial dependence is shown by the square symbol (average difference in proportion male response: 0.08 ± 0.13, *p* = 0.04)

Twelve participants (5 female) were asked to classify a sequence of face images as *male* or *female* (Fig. 1a and Methods). The images were of three types, male, female, and androgynous, constructed from a morphed face space. Despite calibration aiming at 25, 50, and 75% male response for female, androgynous, and male respectively, there remained a tendency to respond male rather than female to androgynous faces (average proportion male response: 0.62 ± 0.14 STD): this is evident from the data points of Fig. 1b, mostly above the 50% male responses (fine dashed lines), for both the ordinate and the abscissae. However, in addition to this constant bias, there is a clear bias that depends on the previous response, as most responses lie above the diagonal: participants responded *male* more often when the previous response was *male* than when it was *female*. That is, there was a significant attractive serial dependence effect (average difference = 0.082 ± 0.13 STD, paired *t*-test *p* = 0.04), where serial dependence is calculated as the distance of the point to the diagonal. The effect varied considerably across subjects, suggesting genuine individual differences, consistent with previous studies [9, 22]. Separating participants based on their gender did not reveal any meaningful difference in overall bias (average proportion male response for female participants = 0.68 ± 0.21 STD, average proportion male response for male participants = 0.58 ± 0.11 STD).

**Fig. 2:**
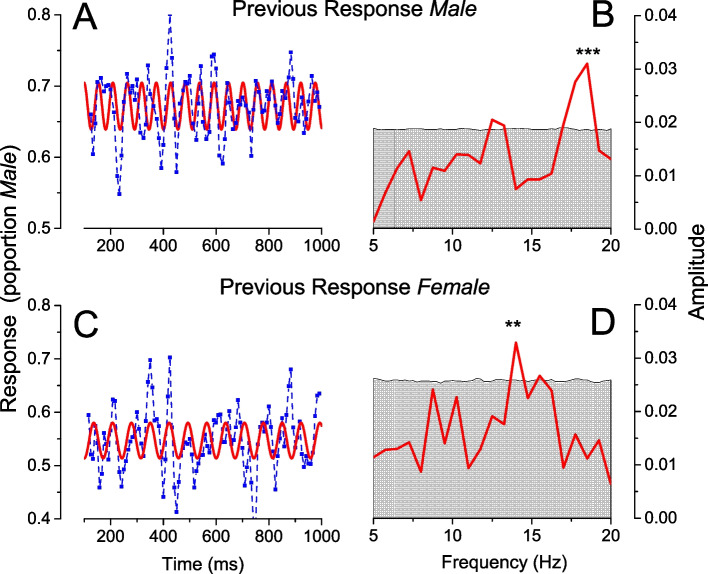
Timecourses and Fourier spectra of response bias. **A** Proportion of “*male*” responses to androgynous stimuli preceded by response *male*, as a function of time after the auditory tone (blue symbols). The red curve shows the most significant sinusoid fit, at 18.5 Hz. **B** Fourier amplitude spectra calculated by single-trial GLM (see Methods) of the data of **A**, showing a single significant peak at 18.5 Hz. **C** Same as **A**, but for stimuli preceded by response *female*, with best fitting sinusoid at 14 Hz. **D** Same as **B**, for stimuli preceded by response *female*. ** denotes *p* < 0.01, *** *p* < 0.001, FDR corrected for all frequencies in the range 5–20 Hz of the surrogate data

As illustrated in Fig. 1a, each trial was initiated with an auditory tone, followed by the face stimulus after an interval ranging from 100 to 1000 ms. This procedure aimed to reveal oscillations in the response, as evidence suggests that salient auditory stimuli (and also motor actions) can reset the phase of endogenous oscillations [23, 24]. Figure 2A and C show the oscillatory biases in responses, plotted as a function of time after the auditory tone, separately for when the previous response was *male* and *female*. In both cases, the responses were not constant, but oscillated over time, faster for preceding *male* than *female* responses. The red curves of Fig. 2A and C show the sinusoidal fit at the peak frequency calculated by a single-trial GLM (Fig. 2B, D). Following a male response, bias oscillated at 18.5 Hz (*p* < 0.001, corrected for all frequencies in the range 5–20 Hz of the surrogate data: see Methods). Following a female response, bias oscillated most strongly at 14 Hz (significance *p* < 0.01). These results replicate the findings of Bell et al. [21], where bias synchronized to voluntary button press (possibly a stronger endogenous reset) oscillated at 18 Hz, for previous male face stimuli, and 13.5 Hz for female face stimuli. The effect was replicated, despite the different reset signal (motor vs auditory), and using response rather than stimuli to calculate serial dependence.

### EEG results (ERP and power analysis)

**Fig. 3:**
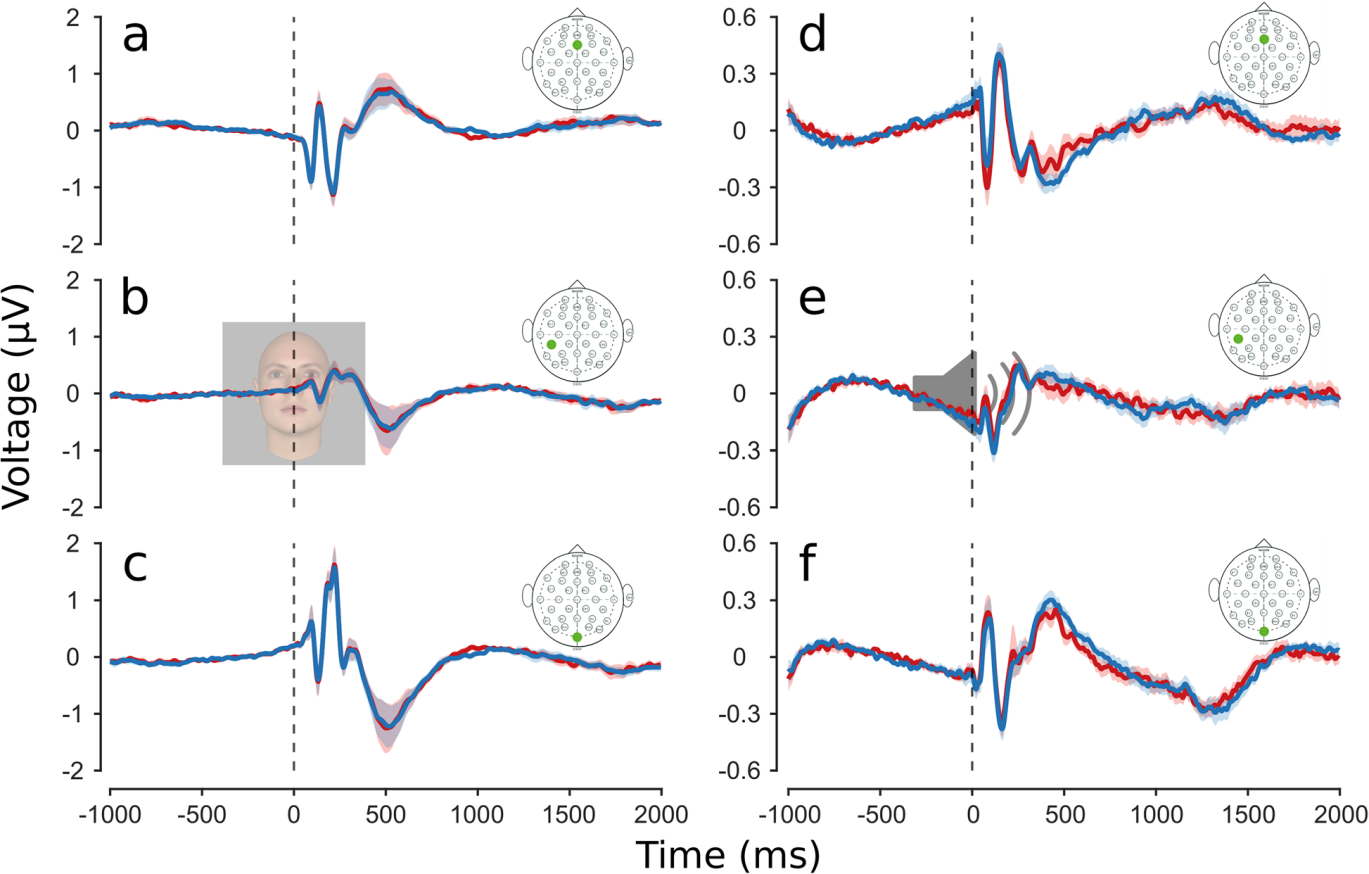
Event-related potentials (ERPs) separated according to previous response. **a**–**c** ERP synchronized to face stimulus presentation of example Fz, CP5, and Oz electrodes. In blue ERP of trials where participants responded male to the previous trial, in red where they responded female. Color shading represents ± standard error of the mean (SEM). **d**–**f** ERP synchronized to audio cue presentation of Fz, CP5, and Oz electrodes

The main goal of this research was to study the neural mechanisms behind serial dependence, recording EEG from participants while they made sequential psychophysical judgments. We first analyzed the event-related potentials (ERPs) for the two conditions “previous response male” and “previous response female” (average result in Fig. 3a–c for electrodes midline frontal (Fz), left central parietal (CP5), and midline occipital (Oz), chosen to illustrate the visual, auditory, and decisional dynamic of the responses, to observe any possible average differences in the visual stimulus response). There were no clear or significant differences between the two conditions, with the two curves virtually superimposed on each other. We also analyzed the responses aligning them to the phase-resetting auditory tone (Fig. 3d–f). In the period before the tone, the two ERPs were always superimposed. In the period after the auditory cue, when the visual stimulus was presented (possibly contaminating the traces), there was a trend of higher beta oscillation (red curves) when the previous response was female, but the difference did not reach statistical significance across subjects. Note that the number of trials in the two conditions are different, being more numerous in the previous response male, given the male-response bias.

We tested for oscillations in the raw EEG signal by calculating the power at 14 and 18.5 Hz in the period − 500 to − 20 ms before auditory cue for each trial, and found no statistically significant difference between trials preceded by male or by female responses.

### Decoding results

Given the lack of significant difference in oscillation power in the raw EEG data, we applied more efficient classification techniques to test whether decoding EEG signals generated by the *current* trial could classify the responses to the *previous* face stimuli. This technique is based on small differences in signal distribution across electrode position. It relies on few assumptions, as all brain activity is used, without selecting electrodes or ROIs. Given the hypothesis that the beta frequencies are related to serial dependance (based on the behavioral oscillations), we filtered the EEG signal in the 14–20 Hz beta range and used the signal power for SVM decoding.

**Fig. 4:**
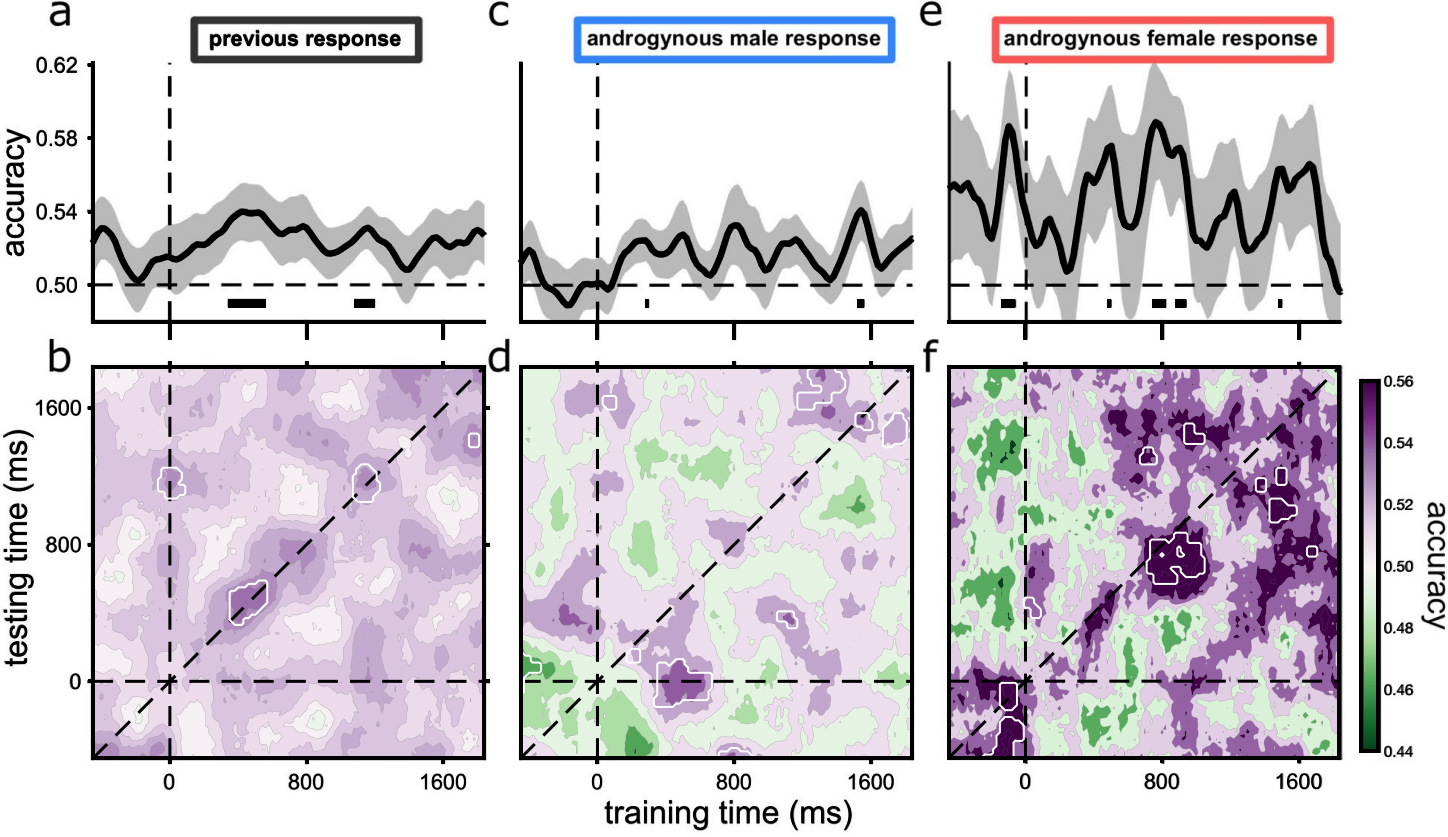
Decoding of previous responses from current-trial EEG activity. In all decoding procedures, classifiers were trained on all trials to distinguish between *previous* response male and *previous* response female, using the current EEG response. The classifiers were tested in three ways: **a** Classifier accuracy as a function of duration after current trial onset (of both training and testing) for all trials of the previous stimuli. **b** Generalization matrix for the same stimuli as in **a**, decoding all possible combinations of training and testing times. White contours surround regions of significant accuracy after cluster correction. Decoding was strongest along the diagonal (which corresponds to **a**), but there were also significant regions of decoding away from the diagonal, suggesting generalization of decoding. **c**, **d** Same as **a** and **b**, but only for trials where the current stimulus was androgynous and the current response was “male.” **e**, **f** Same as **a** and **b**, but only for trials where the current stimulus was androgynous and the current response was “female”

For all the decoding analyses reported in Fig. 4, classifiers were trained and tested to distinguish the responses of the *previous* trial (male versus female), using the *current* EEG traces of 80% of all trials (see Methods for full details), sampled from all three stimulus types (male, female, and androgynous). The remaining 20% of trials were used for testing. Either all trials were used (panels a and b) or a specific subset, defined by the current trial (only androgynous stimuli, response either “male” or “female”).

Figure 4 shows the results: the lower plots show the full generalization matrices as a function of training and testing times, the upper plots the diagonals of those matrices where training and testing times coincide. Figure 4a shows classification accuracy as a function of time after stimulus presentation (of both training and testing), using all trials in the dataset. Accuracy was consistently above chance and reached statistical significance (after multiple comparison cluster correction) between 340 and 560 ms after stimulus onset, and again between 1080 and 1200 ms. This shows that there is information about the *previous* trial in the neural response to the current trial. The late cluster may result from participants having to wait at least 1 s before responding, keeping the information active.

We calculated the temporal generalization matrix of classification accuracy across all training and testing times to distinguish dynamic from static decoding: dynamic coding should result in high accuracy along the diagonal, where testing time intervals are the same as training times; static encoding could result in high accuracy at many points where training and testing did not coincide. Static encoding would imply that the signal of the previous response embedded in the visual response is the same at all intervals. The generalization matrix is shown as a heat map in Fig. 4b. There are four regions where decoding was significantly above chance (within the white-outlined regions of Fig. 4b), two corresponding to those illustrated in Fig. 4a (the diagonal of the matrix), and two extra regions of smaller cluster size, one at 0 training and 1200 ms testing, the other at 1800 ms training and 1400 ms testing. The additional significant regions away from the diagonal are evidence for some generalization for different training and testing times. The classification appears to be mainly dynamic, with decoding best when training and testing times coincide but there seems to be also a static component, consistent with reactivation of a memory trace that does not depend on the current stimulation.

To investigate further the relevance of this trace on participant responses, we considered only trials where the *current* stimulus was androgynous, and further separated them on the basis of response to the current trial (*male* or *female*: Fig. 4c–f). We used the same classifier model as in Fig. 4a and b (using all trials), but tested them on two subsets of data: *current* androgenous stimuli with response *male* and current androgenous stimuli with response *female*, to examine separately decoded signals in trials where participants had higher likelihood of being biased towards one of the two responses based on the presence of the memory trace. In selecting the trials for testing, we used the minimum number of trials in both sets, discarding at random superfluous trails in the previous male response set (the more numerous set). This minimizes possible effects of the male bias in the difference between the two generalization matrices.

The results show higher decoding accuracy of androgynous trials where participants responded *female* (difference in peak accuracy between male and female decoding: 0.06 ± 0.015 SEM, *p* = 0.003, Log (BF_10_) = 1.16). The reason behind the better decoding of female may be related to the general bias of participants towards *male* response, so there is more information in the response *female.* However, other alternatives are possible. Despite matching the overall number of trials for the classifications, for the male responses the trials were randomly resampled between a larger pool of ERP responses, decreasing the overall noise. Despite these differences in accuracy, the highest accuracy again tends to be along the diagonals, but some regions off-diagonal are significant, pointing again to limited generalization of coding and decoding.

**Fig. 5:**
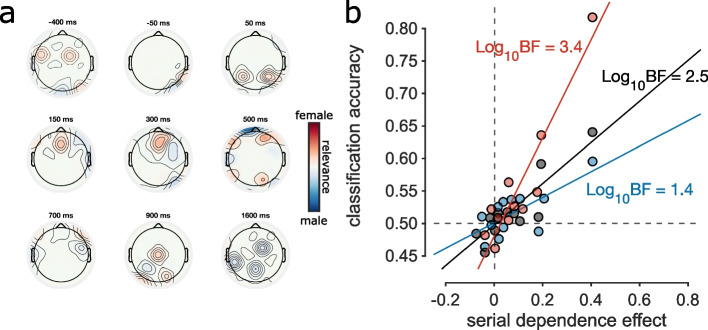
Activation patterns and correlation with behavior. **a** Activation patterns of classification showing the relative weights assigned to electrodes for decoding at example time points. Positive weights (red) indicate that higher power at the location sways classifiers to identify the signal as belonging to the “previous female” class, while negative weights (blue) indicate that higher power sways classification towards “previous male.” Weights are normalized on maximum activation across electrodes and time. **b** Correlation between serial dependence strength and classification accuracy across participants, for the 3 decoding conditions in Fig. 4. Serial dependence strength (abscissa, constant across all 3 conditions) was calculated as the difference between proportion male response with previous male response and proportion male response with previous female response. Classification accuracy was calculated by averaging individual participant accuracy across the diagonal ± 2 points (final precision ± 60 ms) in the window 0–1 s. Decoding tested on all trials in black, on androgynous trials with female response in red, and on androgynous trials with male response in blue. The strength of the correlation is given by Log_10_ Bayes factor, shown near each fit

Activation maps (Fig. 5a) show the electrodes that were most informative for classification at different times. Before stimulus presentation decoding relied on a stable right-occipital dipole and on distributed frontocentral locations. In early perceptual processing (up to ~ 60 ms), the memory signal was classified by activity of occipital electrodes. The signal shifted progressively to frontal locations up to 140 ms, when just frontal electrodes contributed to classification. At 300 ms, the time when decoding of the model reaches statistical significance (after cluster correction), the most informative electrodes of the models are in frontal cortex. At around 500 ms, the middle of the significant interval, parietal and occipital locations also became relevant, remaining relatively stable up to about 1600 ms. Activation maps before stimulus presentation and well after stimulus presentation were quite different, consistent with the lack of generalization for those intervals across training and testing time (Fig. 4b, d, f top-left and bottom-right corner).

### Correlations between decoding accuracy and strength of serial dependence

We tested the correlation between classification accuracy and serial dependence across participants (Fig. 5b). Given no strong evidence of generalization of decoding (Fig. 4b), classification accuracy was averaged over the diagonal window of the temporal generalization matrix (± 2 points along the diagonal corresponding to a precision ± 60 ms), and serial dependence calculated as the difference of proportion male responses on androgynous trials when the previous response was male compared with when it was female. We found a significant positive correlation of serial dependence with average decoding of all trials (black dots,* r* = 0.89, *p* < 0.001, Log_10_BF = 2.5). The correlation of memory trace and serial dependence was stronger for classification accuracy of female-biased trials (red dots, *r* = 0.93, *p* < 0.0001, Log_10_BF = 3.4) compared with male-biased trials (blue dots, *r* = 0.80, *p* = 0.002, Log_10_BF = 1.4). Similar results were also obtained with robust regression with bisquare weights (significant correlation with *p* < 0.00001), and also when outlier participant with serial dependence greater than 0.4 were discarded (significant correlation, *p* < 0.02). Although most participants showed a strong bias towards *male* classification, this bias did not correlate with classification accuracy (Log Bayes factor = − 0.2), neither when considering all trials, or separately for androgynous trials preceded by *male* or *female* responses.

**Fig. 6:**
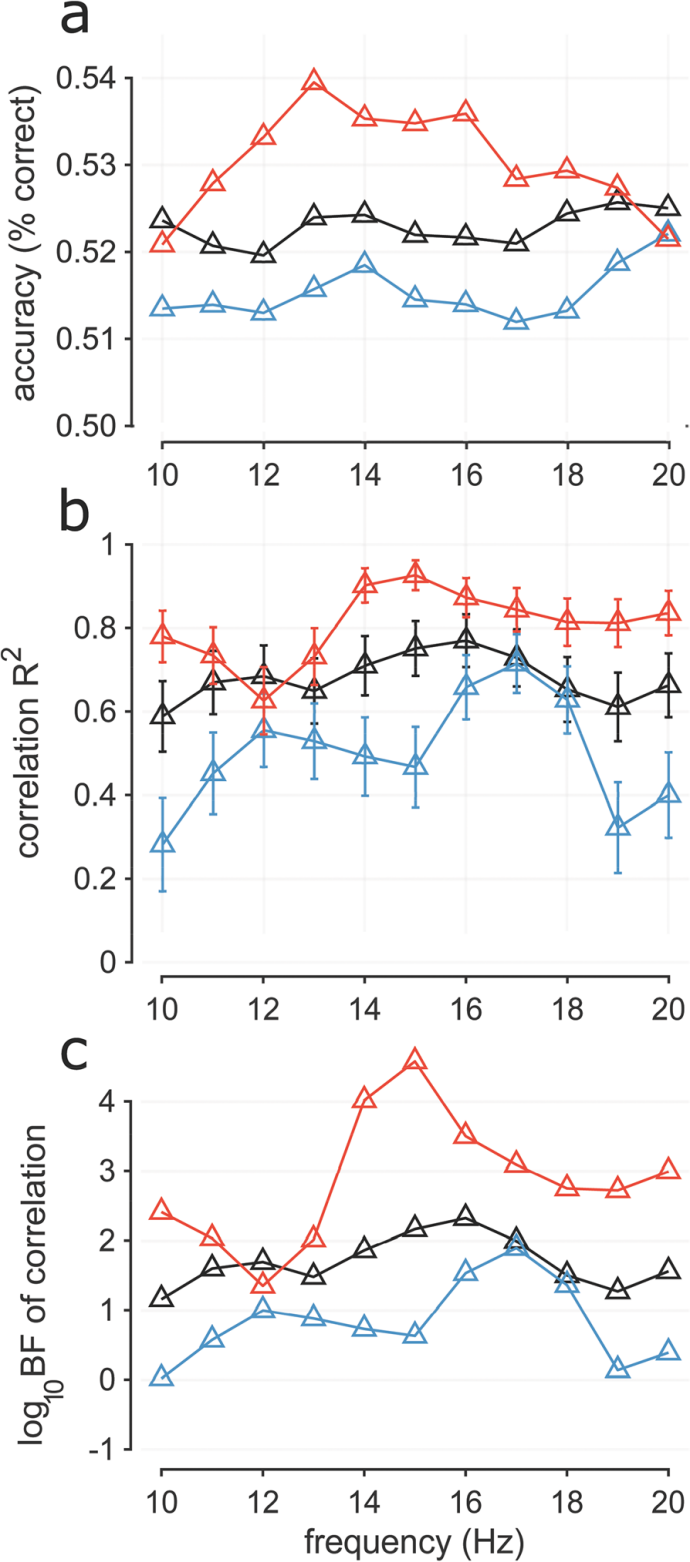
Decoding with bandpass filtered stimuli. **a** Classification accuracy across frequency calculated as average across the diagonal in the window 0–1 s. Classification accuracy was tested on all trials (black), on androgynous trials with female response (red), and on androgynous trials with male response (blue). **b** Squared correlation coefficients (*R*^2^: explained variance) between classification accuracy and serial dependence strength across participants (errors show ± 1 s.e.m.). Classification accuracy calculated as in **a**. The serial dependence effect was calculated as the difference between proportion male response with previous male response and proportion male response with previous female response. **c** Logarithms of Bayes factor of the correlations of **b**, lending statistical support to the stronger correlations at the peaks

To relate the EEG results to the psychophysics, which showed clear beta-frequency oscillations, we repeated the decoding analysis after filtering the EEG into narrow-band windows of 2 Hz, from 4 to 20 Hz. Figure 6a shows how decoding accuracy varied with filter frequency. Considering all androgenous stimuli, average classification was relatively flat across frequency (black dashed line, Fig. 6a). However, confining the analysis to androgynous stimuli with female response shows a shallow peak in decoding around low-beta, 12–18 Hz (red line, Fig. 6). The trend with androgynous stimuli with male response was less clear, but tended to increase over the beta range, peaking at the maximum analyzed, around 20 Hz. This is not inconsistent with the psychophysical results, but not strong support.

As decoding accuracy covaried with the magnitude of serial dependence, showing a strong link between the psychophysics and EEG, we next tested whether the strength of the correlation varied with frequency range. Figure 6b shows how the square of the correlation coefficient (*R*^2^, the variance explained by the correlation) varies with filter frequency. The correlation considering all androgenous trials is again relatively flat, but the correlation for the androgenous stimuli with *female* response shows peaks at around 14–15 Hz, while that for androgenous stimuli with male response peaks at 17 Hz and is lower in value, consistent with the results of Fig. 5b. Considering the 2 Hz bandwidth of the filters, this is qualitatively very similar to the psychophysical results showing peaks at 14 and 18 Hz for previous *female* and *male* responses and those of Bell et al. [21], collected with a slightly different technique on a different sets of participants, based on previous stimuli rather than responses.

To test the robustness of the peaks in correlations, we applied Bayesian statistics, calculating log Bayes factors (difference of the log-likelihoods of the alternate and null hypotheses), shown in Fig. 6c. These log likelihood ratios show clear peaks at 15 Hz for previous *female* stimuli and 17 Hz for *male*. For *male*, LogBFs are less than 1 for most of the range, except for the 3 points around 17 Hz. Differences in the log Bayes factors provide evidence of the likelihood of two alternate hypotheses: 0.5 considered “substantial” evidence, 1 “strong,” and 2 “decisive.” The peak of the red curve (previous female) was more than 0.5 log units higher than both adjacent frequencies, and that for previous male (blue symbols) 0.36 and 0.56 higher than its neighbors. For both curves, the peak was more than 1 log-unit higher than all non-adjacent frequencies, in support of a frequency selectivity. This Bayesian analysis supports the claim that serial dependence related information for decoding previous *female* stimuli is strongest around 15 Hz, but also shows that all beta frequencies contribute to the serial dependence effect; for previous *male* stimuli only frequencies around 17 Hz provide strong support for correlation.

## Discussion

This study investigated the physiological substrate of neural endogenous oscillations instrumental in transmitting predictive information about face-gender. The results show that previous responses leave a lingering EEG trace that can be decoded from the activity of the next stimulus. We report strong correlations between the strength of behavioral serial dependence effects and the strength of neural classification of previous responses. Importantly, the correlations peak at similar frequencies to those identified in behavioral analysis, both in previous research [21] and here, suggesting that perceptual representations of recent experience may be encoded within the spectral structure of neural oscillations.

Since classifiers were trained on averages of single-trial EEG amplitude envelopes, decoding could rely on both phase-locked and non-phase-locked information. The method is very robust against ringing of filters [25], as shown by a large literature of “local energy models” applying these algorithms to in image segmentation [26, 27], making it suitable for application with very narrow band filters, as used in brain-machine interfaces. Temporal generalization maps reveal sparse regions of significant decoding of the previous response, but exploring the distributions of accuracy, especially for androgynous trials with female response, suggests that regions of high accuracy may be distributed over the entire temporal matrix, rather than being confined to the diagonals where training and testing time coincide. This qualitative interpretation would be consistent with a memory signal that is relatively stable in time, with discrete evolution at specific time points [28], where it reaches significance. Decoding accuracy was strongest for androgynous trials with female response and weakest for androgynous trials with male response where there was solid evidence for significance only between 16 and 18 Hz. The simplest interpretation for this difference may be related to the overall male bias of participants, so the female response was more informative, but there is no firm evidence for this suggestion.

We also looked at the raw EEG, synchronized to either the stimulus presentation or to the audio cue. On inspection, oscillations in the trace were visible, especially for trials when participants responded “female.” However, the oscillations did not survive standard tests for significance, so must be considered anecdotal. Clearly, the classification approach was more robust and efficient.

We chose to examine the effect of perceptual history by training classifiers to distinguish the responses of the *previous* trial (male versus female), using all the *current* EEG traces, and testing them on the same task, either on all trials or a specific subset defined by the current stimulus. Obviously, this was not the only way to proceed. We could have trained on the current response and tested on the previous (or vice versa), as we did in a previous publication [29]. However, with the current data set, we found this combination gave less robust results, so we chose to continue training and testing the model on the previous responses. Note that the fact we could train a model by labeling the current EEG with previous responses demonstrates unequivocally the presence of a lingering neural signal from the previous neural activity, which was our main goal. Many other combinations of training and testing combinations would have been possible using current or previous EEG, current or previous responses (or stimuli), etc., but we chose the simplest, most robust, and most informative conditions, after piloting the data.

In our previous study [21] on face-gender discrimination, we showed an oscillatory dependence on the previous *stimulus*, at frequencies very similar to those observed here. In this study, we chose to use responses rather than stimuli as class labels for the classification studies, for several reasons. Firstly, half the stimuli were androgynous, neither male nor female, and therefore uninformative as stimuli: yet the response to those stimuli is very informative, reflecting an internal state. Secondly, as responses to male and female stimuli were about 75% correct, stimuli and responses are highly correlated, hard to disentangle. We are aware of (and have contributed to) the ongoing debate on whether serial dependence acts on early perception or on later decision-making processes [5, 28, 30–34], and whether it can manifest as attractive or repulsive neural signals [18, 34–36], depending also on the dynamics of the effect. However, responses do not represent only late decision stages, but the internal neural state of the participant [37], so the choice of using responses does not speak to this issue. Inspection of the activation patterns of decoders during the interval where significant decoding occurred shows that classification relies mainly on frontal electrodes, migrating to occipital electrodes in the following 100 ms, supporting the idea of a feedback decisional signal to early visual cortex for serial dependance. Consistent with rapid activation of early visual cortices reported in previous studies on serial dependence [32, 38–40], we also observed activation of occipital areas as early as 50 ms after face presentation (earlier than we would expect for bottom-up visual processing) suggests that the representation of the prior may be already embedded in visual cortices, possibly in the form of synaptic gain changes [41–43]. However, caution is needed, as decoding was not statistically significant in this time interval.

Lastly, the correlation between neural signals and behavior in frequency-filtered signals reinforces the behavioral evidence for beta oscillations being instrumental in the behavioral bias. Interestingly, the methods based on the envelopes of EEG signals used here are also used to analyze phase coherence between the various frequency components of the response and endogenous rhythms. The frequencies of peak decoding may correspond to the frequency bands where phases are more coherent [26, 27], as happens during phase resetting after sensory or action cues. This is the same mechanism hypothesized for the interpretation of behavioral oscillations, providing a general framework to relate the behavioral oscillations of Fig. 2 with the peak decoding of Fig. 6.

Several recent studies have employed classification techniques to reveal traces of previous trials, suggesting that perceptual experience lingers as an activity-silent trace (a change in cellular mechanisms, such as strengthening of synapses or gain of particular circuitry), which is then reactivated with new stimulation [29, 41, 42, 44]. In these paradigms, classification accuracy is at chance level before the presentation of a new stimulus, with significant decoding only after presentation. However, other researchers have questioned the activity-silent trace hypothesis. Runyan et al. [45] reported replay of neural activity during rest, which may be related to ongoing consolidation processes that help to strengthen memories over time. Stokes et al. [46] also challenge the idea, suggesting that perceptual learning is supported by changes in neural connectivity and plasticity. We have previously supported the activity-silent trace hypothesis [29], finding that classification accuracy (for spatial frequency discrimination) was not significant before new stimuli were presented. However, the temporal generalization matrices of this study (Fig. 3) for the female bias condition show that the trace is sometimes present before the presentation of a new stimulus, and as mentioned before, this activity is not related to filter ripples. One possibility is that these hot spots result from a lingering trace from the previous stimulus [33], supported by the fact that classifiers trained before time 0 do not accurately classify previous traces after time 0, and vice versa. This suggests that there is a distinct change in the signal when a new stimulus is presented, supporting the action of a silent memory signal.

It is widely acknowledged that observers enhance efficiency by using past information to anticipate future sensory input. The connection between behavior and decoding of previous responses was notably pronounced when limiting the neurophysiological signal to low-beta frequencies, where the behavioral bias oscillated at frequencies that depend on the previously perceived gender. It has been established that low-frequency oscillations contribute to the transmission of predictive information, akin to the concept of perceptual echoes advanced by VanRullen and Macdonald [47]. Ho et al. [9] showed that auditory stimuli oscillated within the alpha range (~ 9 Hz) at distinct phases when presented to either the left or the right ear. Using a similar paradigm to the present study, Bell et al. [21] demonstrated that following the observation of a specific stimulus, whether male or female, the inclination to perceive an androgynous face as female or male oscillated respectively at 13.5 or 17 Hz. Considerable evidence details how bias oscillates at specific frequencies, as observed in visual orientation discrimination [48], trans-saccadic location discrimination [49], and audiovisual temporal judgment [50]. For more intricate perceptual functions, like face-gender discrimination, it is possible that prior expectations require a coding mechanism involving multiple frequency channels. The literature suggests that beta oscillations may play a role in processing local features [51, 52]. It is conceivable that various frequencies of beta oscillations might explain the response patterns elicited by female or male stimuli, as well as more complex stimuli in general. The arrangement of local facial features could potentially clue the interpretation of faces as masculine or feminine.

## Conclusions

Overall, our results suggest that recent experience of face-gender is represented in low-frequency spectral components of intrinsic neural oscillations (low-beta 14–20 Hz). The strength of the active trace correlates with the strength of serial effects in behavior, especially in the low-beta range, suggesting that our signal may reflect the underlying neural substrate of the attractive effect. These results suggest that recent experience lingers in perceptual cortices and changes with new stimulation, possibly becoming strengthened. The strong correlation between decoding accuracy and the strength of serial dependence further suggests that these oscillatory signals are highly instrumental in the transmission of internal models, within the predictive coding framework [6, 53]. Our results corroborate the intuition of Bastos et al. [54] that in a hierarchical predictive coding framework, low-frequency neural oscillations in encephalography (4–22 Hz range) are a good candidate for top-down internal models.

## Methods

### Participants

Twelve healthy adults (7 females, age range 20–29 years, mean = 24.8 years, SD = 2.7 years) participated in the experiment with monetary compensation (€10/h). All participants had normal or corrected-to-normal vision and gave written informed consent. The experimental design was approved by the local regional ethics committee (*Comitato Etico Pediatrico Regionale—Azienda Ospedaliero-Universitaria Meyer—Firenze*, permit number 3/2011, reapproved 23/11/2021) and is in line with the Declaration of Helsinki for ethical principles for medical research involving human subjects (DoH-Oct2008). We did not perform a formal power analysis to determine participant number but, based on our previous experience with decoding EEG [29] and also psychophysically measured oscillations of face-gender [21], we reasoned that 12 should be sufficient.

### Stimuli and apparatus

The experiment was conducted in a quiet dark room, where participants sat in a comfortable chair with head rested on a chinrest. The stimuli were presented on a Display + + LCD Monitor (Cambridge Research Systems, 120 Hz, 1920 × 1080 resolution), gamma corrected, 70 cm from the eyes, mean gray screen luminance equal to 50 cd/m^2^. Face stimuli were a subset of images taken from Bell et al. (2020). They were originally generated in FaceGen Modeller 3.5.3 and saved as high-resolution 2D gray scale image (6.6° × 6.6°). The faces were white, mid-twenties, with gender neutral coloring, shape, and typical asymmetry. We performed a preliminary response-balancing procedure on 4 naïve observers who did not participate in the experiment (600 trials each, 25 face identities). We selected 15 face identities based on mean response deviating no more than ± 10% from target accuracies (75% male response for male faces, 50% male response for androgynous, 25% male response for female). The phase-resetting auditory stimulus was a 16 ms, 900 Hz pure tone (80 dB sound pressure level at the ear, 44,100 kHz sampling frequency) delivered through 2 loudspeakers besides the monitor, following [55].

### Procedure

Participants fixated a white dot at the center of the screen, which was present for the whole duration of the experiment, except during presentation of face stimuli. Each trial began with the auditory stimulus. After a random interval ranging uniformly from 100 to 1000 ms (at 120 Hz sampling frequency, the monitor refresh rate), one of the 45 faces was presented for 17 ms (two frames). Participants were instructed to wait at least 1 s before responding, indicating whether the presented face seemed male or female (by pressing the left or right arrow keyboard keys). Response configuration (association of arrows with gender) was randomized between participants and switched halfway through the experiment. After button press, a new trial started after an interval ranging uniformly from 800 to 1100 ms, so that the auditory stimulus presentation was not easily predictable. Each participant completed 1215 trials.

### EEG acquisition and preprocessing

EEG data were collected with a Nautilus Research headset (g.tec) at a sample rate of 250 Hz with no online filtering. The data were referenced online to a unilateral electrode placed behind the left ear. Activity was measured from 32 gel-based active electrodes (g.LADYbird technology) arranged according to the 10/20 system. Impedance was kept below 50 kΩ.

Offline EEG preprocessing was performed in MATLAB (MathWorks®) with custom code. EEG data were referenced to the common average reference and filtered with a FIR bandpass filter (Chebyshev window, 128th order, stopbands 1 Hz and 35 Hz, sidelobe magnitude factor 50 dB). To avoid decoding ripple artifacts in decoding associated with steep cutoff filters [25], we pre-processed all EEG traces for decoding with an energy envelope (see section Data analysis—EEG time-domain and power analysis). For the main data analysis of decoding, epochs were extracted aligned to stimulus presentation, comprising a segment of data from − 500 to 1800 ms after the stimulus. Epochs were visually inspected for motor artifacts and wireless failure of signal transmission (manual rejection of 0.8% ± 0.6% STD of data across subjects). Ocular artifacts were removed through blind source separation with ICA decomposition [56].

### Data analysis—psychophysics

We analyzed individual responses to androgynous face identities, calculating the proportion of “*male*” responses, separately for previous “*male*” and “*female*” responses. We removed from all analyses trials where response latencies were shorter than 1 s or longer than 3 s (average response latency 2.2 s; CI95 = 1.6–2.7 s). After EEG artifact and response time removal we obtained 1010 ± 14 (std dev) trials per subject. To assess whether previous responses changed gender discrimination bias, we compared by *t*-test proportion “*male*” responses to androgynous trials when previous response was *male* to when previous response was *female*).

To measure oscillations in face-gender behavioral bias, we applied single-trial analysis to aggregate data from all participants, including all the trials that survived the EEG artifact rejection procedure. Single-trial analysis is immune to problems of curve-fitting and binning (ref [57]: but see also reviewer criticism at https://www.nature.com/articles/s41562-022-01364-0#MOESM3 and [58, 59]). The general linear model (GLM) analysis weighted each single trial with the following model:

1 $$y={\beta }_{0}+{\beta }_{1}\text{sin}(2\pi ft)+{\beta }_{2}\text{cos}(2\pi ft)$$

where *y* is participant response (1 for male, 0 for female), *t* is SOA (audio to face) in seconds, *f* is a fixed frequency ranging from 5 to 20 Hz at 0.75 Hz intervals, and *β*_0_, *β*_1_, and *β*_2_ are free parameters. We separated responses to current androgynous stimuli based on previous response (male or female), and fit the above GLM, calculating the amplitude of the sinusoidal fit to each frequency in the range 5–20 Hz. To assess statistical significance, the surrogate data generated by shuffling the responses (10,000 permutations) were analyzed with the same GLM obtaining a distribution of amplitudes across frequencies under the null hypothesis. Frequencies with amplitudes over the 95th percentile of this distribution were candidates for statistical significance, after false discovery rate correction [60]. For illustration purposes only (Fig. 2), we show the responses binned in synchrony with the tone at 8.3 ms intervals, with a running average of 3 consecutive bins (weights: 0.305, 0.39, 0.305).

### Data analysis—EEG time-domain and power analysis

We calculated event-related potentials (ERPs) synchronized to face stimulus presentation, separately for the two conditions “previous male” and “previous female” responses. Grand-average ERPs were calculated by averaging single-participant ERPs normalized (*z*-scores) to a baseline window in the interval from − 500 to − 100 ms with respect to stimulus presentation.

We classified previous (*n* − 1) responses from the EEG activity elicited by the current (*n*) trial. Before classification, we filtered the entire time series of 111 ± 18 min across participants, in a low-beta range (14 to 20 Hz) (given the hypothesis from behavioral data [21]), and calculated the amplitude of the local EEG signal envelope as the norm of the complex vector where the real part is the EEG signal and the imaginary part is its Hilbert transform computed for all the filtered EEG signals (Fig. 3). For the second decoding procedure (Fig. 6), we filtered narrowly around frequencies from 4 to 20 Hz at 1 Hz steps (Type II Chebyshev window, 1024th order, passband 0.5 Hz above and below the selected frequency, stopband 1 Hz above and below the selected frequency, sidelobe magnitude factor 50 dB). We analyzed only the signals in the band greater than 10 Hz as this band has more than 9 periods in the interval of stimulus presentation, robust to sudden transient signal changes in any electrodes. We down-sampled trials to 50 Hz, obtaining 150 time points from − 500 to 1000 ms, synchronized to face stimulus presentation.

Decoding of single-trial EEGs is usually not feasible, given the low S/N ratios with short stimulus exposure (16.6 ms), so it is recommended to average trials of the same categories. In our previous study [29], we averaged over 7 trials; here, given that only two response classes (male/female) were present, we averaged over only 5 trials of the same class (as in ref [61]), obtaining our final classification samples. We used binary support vector machine (SVM) classifiers with linear kernel (the MATLAB *fitcsvm* function), using the 32 electrode locations as features for classification.

For all the decoding analyses, classifiers operated on the *current* EEG signals and were always trained and tested on classification of the participant responses of the *previous* trials (male versus female). The training phase employed all three stimulus types (male, female, and androgenous) and all trials, while the testing phase used either all trials, or a subset (current androgynous trials to which observers responded “male” and current androgynous trials which observers responded “female”). For each participant, and on each iteration, samples were split into 5 folds, with a 4:1 training to testing ratio (196 ± 14 SD samples in the training set per observer). The procedure was repeated 5 times, rotating folds for testing. The whole decoding procedure was repeated 100 times, generating new samples every time by averaging random sets of 5 trials together. This allowed us to minimize lucky splits of the data and assess accuracy by averaging over a large number of guesses (24,519 ± 141 SD guesses per observer). Each of these iterations was performed on a balanced pool of trials, equal to the minimum trials (multiple of five) in the two classes. Although this lowered the classification pool, it ensured that the two classes always had comparable signal-to-noise ratio.

We assessed temporal generalization by testing classifiers across all time points, obtaining a matrix of accuracy across training and testing times per participant. We averaged accuracy across participants and extracted matrix diagonals to show the dynamics of previous response signals. Statistical power was defined by *t*-tests between subjects against 50% accuracy. To correct for multiple comparisons, we implemented a strict cluster correction based on surrogate data permuting image labels, following the procedure introduced by Bae and Luck [41], who introduced a conservative criteria for a cluster reaching significance. We used the same model trained on real data but tested it on surrogate (label-shuffled) data, resulting in a conservative criterium for statistical significance. We permuted class labels at the level of testing, regenerating the 5 average signals and keeping the same label shuffle for all temporal series to keep intact the temporal correlation of the EEG signal, as in [41]. For each participant, we generated 2000 temporal generalization matrices from surrogate data. Each of the 2000 matrixes were averaged across participants to calculate a *t*-test against 50% accuracy, generating a distribution of size of clusters of significant decoding. From each of the 2000 iterations, only the maximum cluster size was retained. Calculating the 95th percentile of this distribution of maximum cluster size from surrogate data, we obtained a threshold of cluster size. To correct for cluster size, all clusters of significant *t*-test across subjects in the real data smaller than the identified thresholds were deemed non-significant.

Activation patterns show the relative relevance of electrode sites for classification, giving qualitative insight on model dynamics. Activation patterns were calculated as follows:

2 $$A=\beta *{X}^{T}$$

where *β* are classifier coefficients and *X*^*T*^ is the transposed matrix of EEG signal of tested data.

## Data Availability

All data generated or analysed during this study are included in this published article and the publicly available repositories: https://figshare.com/articles/dataset/Data_for_Serial_Dependence_in_face-gender_classification_revealed_in_low-beta_frequency_EEG_Ranieri_Burr_Bell_and_Morrone/29245106.

## Abbreviations

EEG: Electroencephalogram
ERP: Event-related potential
Fz: Midline frontal
CP5: Left central parietal
Oz: Midline occipital
ms: Millisecond
Hz: Hertz (cycles/s)
STD: Standard deviation
Log_10_BF: Base 10 logarithm of Bayes factor
